# Synthesis and Evaluation of the Antidepressant-like Properties of HBK-10, a Novel 2-Methoxyphenylpiperazine Derivative Targeting the 5-HT_1A_ and D_2_ Receptors

**DOI:** 10.3390/ph14080744

**Published:** 2021-07-29

**Authors:** Kinga Sałaciak, Natalia Malikowska-Racia, Klaudia Lustyk, Agata Siwek, Monika Głuch-Lutwin, Grzegorz Kazek, Justyna Popiół, Jacek Sapa, Henryk Marona, Dorota Żelaszczyk, Karolina Pytka

**Affiliations:** 1 Department of Pharmacodynamics, Faculty of Pharmacy, Jagiellonian University Medical College, Medyczna 9, 30-688 Krakow, Poland; kinga.salaciak@doctoral.uj.edu.pl (K.S.); malikow@if-pan.krakow.pl (N.M.-R.); klaudia19.lustyk@uj.edu.pl (K.L.); grzegorz.kazek@uj.edu.pl (G.K.); jacek.sapa@uj.edu.pl (J.S.); 2 Department of Behavioral Neuroscience and Drug Development, Maj Institute of Pharmacology, Polish Academy of Sciences, 12 Smetna St., 31-343 Krakow, Poland; 3 Department of Pharmacobiology, Faculty of Pharmacy, Jagiellonian University Medical College, Medyczna 9, 30-688 Krakow, Poland; agat.siwek@uj.edu.pl (A.S.); monika.gluch@uj.edu.pl (M.G.-L.); 4 Department of Pharmaceutical Biochemistry, Faculty of Pharmacy, Jagiellonian University Medical College, Medyczna 9, 30-688 Krakow, Poland; justyna.popiol@uj.edu.pl; 5 Department of Bioorganic Chemistry, Faculty of Pharmacy, Jagiellonian University Medical College, Medyczna 9, 30-688 Krakow, Poland; hen.mar@interia.pl

**Keywords:** depression, 2-methoxyphenylpiperazine, forced swim test, chronic corticosterone administration, 5-HT_1A_ receptor, D_2_ receptor

## Abstract

The increasing number of patients reporting depressive symptoms requires the design of new antidepressants with higher efficacy and limited side effects. As our previous research showed, 2-methoxyphenylpiperazine derivatives are promising candidates to fulfill these criteria. In this study, we aimed to synthesize a novel 2-methoxyphenylpiperazine derivative, HBK-10, and investigate its in vitro and in vivo pharmacological profile. After assessing the affinity for serotonergic and dopaminergic receptors, and serotonin transporter, we determined intrinsic activity of the compound at the 5-HT_1A_ and D_2_ receptors. Next, we performed behavioral experiments (forced swim test, tail suspension test) to evaluate the antidepressant-like activity of HBK-10 in naïve and corticosterone-treated mice. We also assessed the safety profile of the compound. We showed that HBK-10 bound strongly to 5-HT_1A_ and D_2_ receptors and presented antagonistic properties at these receptors in the functional assays. HBK-10 displayed the antidepressant-like effect not only in naïve animals, but also in the corticosterone-induced mouse depression model, i.e., chronic administration of HBK-10 reversed corticosterone-induced changes in behavior. Moreover, the compound’s sedative effect was observed at around 26-fold higher doses than the antidepressant-like ones. Our study showed that HBK-10 displayed a favorable pharmacological profile and may represent an attractive putative treatment candidate for depression.

## 1. Introduction

Due to the COVID-19 pandemic, the prevalence of depression dramatically increased—up to three times as many adults are reporting symptoms of depression now in comparison to last year [1]. However, the available treatment strategies are not always 100% effective in all depressed patients [2]. Moreover, patients often discontinue the treatment as most currently used antidepressants show their clinical effect after a few weeks of treatment and elicit many side effects [3,4,5]. Eventually, the disease course is severe and unstable, often leading to suicide [6]. Therefore, there is an urgent need to search for novel antidepressants with higher efficacy and limited side effects.

Numerous studies examined the role of the serotonergic system in depression and its treatment [7]. Scientists particularly point at the 5-HT_1A_ receptor and its essential role in mood-related disorders [8]. Both human and animal studies demonstrated a reduced level of 5-HT_1A_ receptors located postsynaptically in the prefrontal cortex in depressed individuals [9,10]. On the other hand, the increased expression of 5-HT_1A_ autoreceptors was responsible for the resistance to antidepressant treatment [11]. Considering these findings, the antidepressant-like activity requires the activation of postsynaptic, cortical 5-HT_1A_ heteroreceptors, not presynaptic autoreceptors [12,13].

**Figure 1:**
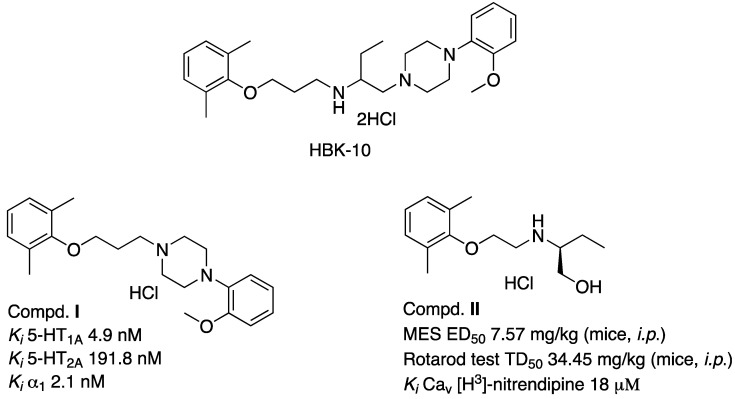
The structure of HBK-10 and the reference compounds **I** and **II** [14,17].

A premise to the synthesis of the HBK-10 were results of the central nervous system and cardiovascular studies of two other compounds from the group of 2,6-dimethylphenoxyalkyl derivatives, namely: 1-[3-(2,6-dimethylphenoxy)propyl]-4-(2-methoxyphenyl)piperazine hydrochloride (**I**) and (*S*)-{2-[(2-(2,6-dimethylphenoxy)ethyl]amino}butan-1-ol hydrochloride (**II**) (Figure 1). Compound **I** showed antidepressant- and anxiolytic-like properties and high affinity toward serotoninergic receptors (i.e., 5-HT_1A_, 5-HT_2A_, 5-HT_7_) [14,15]. It also demonstrated a high affinity for α_1_-adrenoceptor and adrenolytic activity (Figure 1) [16]. In contrast compound **II** showed a promising anticonvulsant effect in in vivo studies [17]. It displayed anti-MES activity with a protective index (TD_50_/ED_50_) of 4.55 and the affinity for the voltage-sensitive calcium channels (Figure 1). The racemic form of compound **II**, containing (*R/S*)-2-amino-1-butanol moiety, was less active in the same study, but was not neurotoxic at the dose of 30 mg/kg (mice, *ip*).

Our extended research, in agreement with other studies, proved that compounds with 2-metoxyphenylpiperazine fragment bind strongly to 5-HT_1A_ receptor and show promising pharmacological profile in animals, including anxiolytic-like, procognitive, and antidepressant-like activity without notable undesirable effects [18,19,20]. Moreover, the compounds were active not only in naïve animals, but also reversed behavioral and biochemical changes in depression model induced by chronic corticosterone injections [21,22]. As not only our studies suggest that 2-metoxyphenylpiperazine fragment determine the high affinity towards 5-HT_1A_ receptors [23], we decided to synthesize a novel 2-metoxyphenylpiperazine derivative and investigate its activity using in vitro and in vivo assays.

Here, we described the synthesis of the 2-metoxyphenylpiperazine derivative (HBK-10) and its pharmacological activity. After the preliminary in vitro safety studies, we investigated the compound’s affinity toward serotonergic 5-HT_1A_, 5-HT_2A_, 5-HT_6_, and 5-HT_7_ receptors, serotonin transporter (SERT), and dopaminergic D_2_ receptor. Subsequently, we investigated the intrinsic activity of the tested compound at 5-HT_1A_ and D_2_ receptors. Next, we evaluated the antidepressant-like properties in vivo using the forced swim test (FST) and the tail suspension test (TST) in mice. Then, we utilized the corticosterone-induced mouse depression model to investigate whether the compound shows an antidepressant-like effect upon a chronic administration. Moreover, we assessed the possible mechanism of action as well as the in vivo safety profile of the tested compound.

## 2. Results

### 2.1. Chemistry

**Scheme 1:**
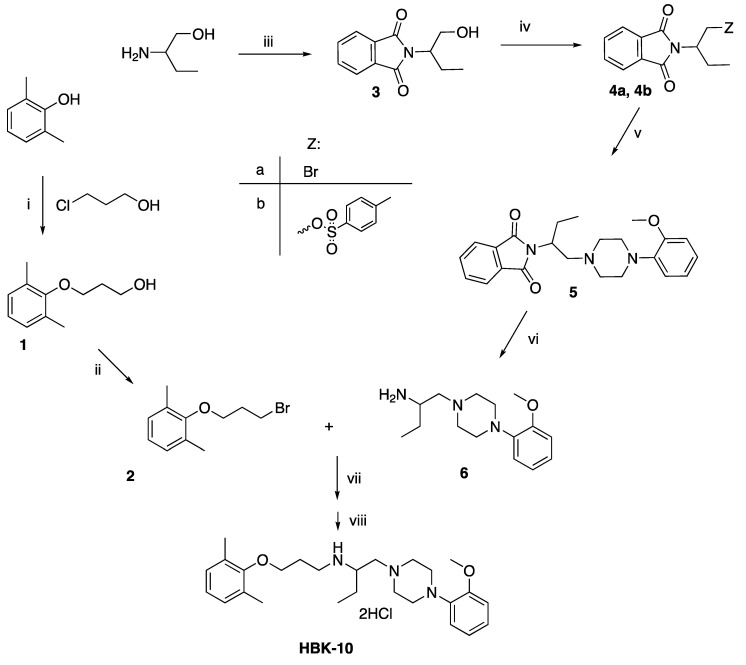
Synthetic route to HBK-10. Reagents and conditions: (i) 3-chloro-1-propanol, K_2_CO_3_, acetone, TEBA, reflux; (ii) PBr_3_, ca 80 °C; (iii) phtalic anhydride, 120 °C; (iv) (a) PBr_3_, ca 80 °C; (b) *p*-toluenesulfochloride, pyridine, toluene, reflux; (v) (a) 1-(2-methoxyphenyl)piperazine, K_2_CO_3_, *n*-butanol, reflux; (b) 1-(2-methoxyphenyl)piperazine, K_2_CO_3_, 2-methoxyethanol, reflux; (vi) 98% hydrazine, ethanol, reflux; (vii) K_2_CO_3_, toluene, reflux; (vii) gaseous HCl, acetone:ethanol (5:1), rt.

The target compound—HBK-10—was synthesized according to the reaction pathways illustrated in Scheme 1. The first key intermediate 2 was obtained from 2,6-dimethylphenole in reaction with 3-chloro-1-propanole giving 3-(2,6-dimethylphenoxy)propan-1-ol (**1**) that was subsequently transformed into 2-(3-bromopropoxy)-1,3-dimethylbenzene (**2**) in reaction with PBr_3_ [14]. The second key intermediate **6** was obtained in a multistep procedure. The first racemic 2-amino-1-butanol was reacted with phtalic anhydride to give 2-(1-hydroxybutan-2-yl)isoindoline-1,3-dione (**3**) [24]. The relatively low yield of 2-(1-(4-(2-methoxyphenyl)piperazin-1-yl)butan-2-yl)isoindoline-1,3-dione (**5**) obtained by bromination of **3** to get 2-(1-bromobutan-2-yl)isoindoline-1,3-dione (**4a**) and further amination with 2-methoxyphenylpiperazine in butanol, prompted us to check another method. Compound **3** reacted with *p-*toluenesulfonyl chloride to yield the 2-(1,3-dioxoisoindolin-2-yl)butyl *p-*toluenesulfonate (**4b**). Amination of **4b** with 2-methoxyphenylpiperazine gave the slightly higher yield of **5**. Hydrazinolysis of the compound **5** with hydrazine hydrate afforded the 1-(4-(2-methoxyphenyl)piperazin-1-yl)butan-2-amine (**6**), the key intermediate substrate, that was used in further step without purification. Then the 2-(3-bromopropoxy)-1,3-dimethylbenzene (**2**) reacted with 1-(4-(2-methoxyphenyl)piperazin-1-yl)butan-2-amine (**6**) to give the final compound HBK-10, namely *N*-(3-(2,6-dimethylphenoxy)propyl)-1-(4-(2-methoxyphenyl)piperazin-1-yl)butan-2-amine. Subsequently it was transformed into dihydrochloride salt, for biological and pharmacological studies.

### 2.2. In Vitro Cytotoxicity and Metabolic Stability

To investigate the lack of hepatotoxicity of HBK-10 at 25 µM concentration, we used HepG2 cells. Viability was measured using 3-(4,5-dimethyl-thiazol-2-yl)-2,5-diphenyltetrazolium (MTT) assay. We chose 25 µM since such concentration was used in metabolic stability studies. Our results show that HBK-10 did not modify HepG2 cells viability at the tested concentration (99%) (Table 1).

Next, the target compound was incubated with mouse liver microsomes in the presence of an NADPH regenerating system. In the initial screening phase, the percentage of the parent compound recovered after 30 min of incubation was calculated (Table 2). This data was compared to the literature value for buspirone. The recovery percent of buspirone after 30 min of incubation with MLMs was lower than 1% and the compound HBK-10 value of recovery percent of parent compound after the same time was more than 100-fold higher (36%). Then, we assessed the half-life and the intrinsic clearance in vitro of HBK-10 and compared it with literature data for aripiprazole and imipramine (Table 2). HB Clint values in microsomal model (MLMs) were higher than the one estimated for aripiprazole [25] and lower than the one calculated for imipramine by Singh [26].

### 2.3. In Vitro Pharmacology

#### 2.3.1. HBK-10 Showed High Affinity for Serotonergic 5-HT_1A_ and Dopaminergic D_2_ Receptors

The radioligand studies revealed that HBK-10 compared with the reference compound, serotonin, exhibited a high affinity for serotonergic 5-HT_1A_ receptors (Table 3). The compound also showed a relatively high affinity for D_2_ receptors, a moderate affinity for 5-HT_2A_ receptors, and very low for 5-HT_6_, 5-HT_7_, and serotonin transporter (Table 3).

#### 2.3.2. HBK-10 Antagonized 5-HT_1A_ and D_2_ Receptors

**Figure 2:**
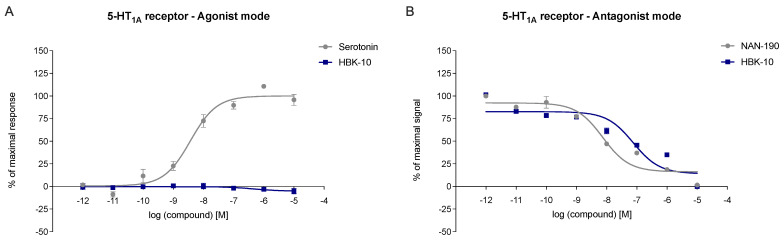
The activity of HBK-10 at 5-HT_1A_ receptors in in vitro functional assays (agonist mode: Panel (**A**); antagonist mode: Panel (**B**)). Graphs represent dose–response curves for functional activity of the compound in inhibition of cAMP production assay.

Since among the tested receptors/transporters, HBK-10 showed the highest affinity for the 5-HT_1A_ receptor, we investigated its intrinsic activity in vitro. Although the 5-HT_1A_ receptor couples to a broad panel of second messengers, it primarily inhibits adenylate cyclase [8]. Thus, we evaluated the influence of HBK-10 on cAMP production using CHO-K1 cells expressing human serotonin 5-HT_1A_ receptor. Contrary to the reference compound, serotonin, HBK-10 did not inhibit cAMP formation, but it showed antagonistic properties in this assay (Figure 2). HBK-10 demonstrated a 10-fold lower equilibrium dissociation constant for a competitive antagonist (K_b_) value compared with the reference compound, NAN-190 (Table 4).

**Figure 3:**
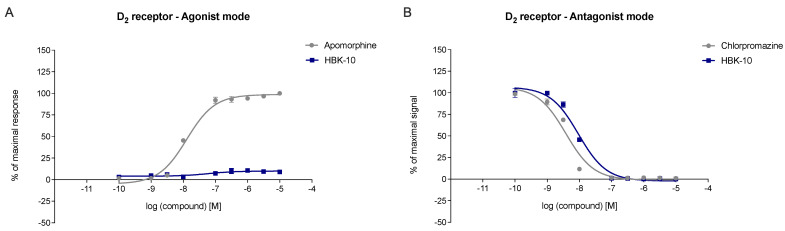
The activity of HBK-10 at D_2_ receptors in in vitro functional assays (agonist mode: Panel (**A**); antagonist mode: Panel (**B**)). Graphs represent dose–response curves for functional activity of the compound in calcium mobilization assay.

Our radioligand studies demonstrated that HBK-10 had a relatively high affinity for the dopamine D_2_ receptor. Therefore, using in vitro functional assay, we next investigated the type of the interaction with this receptor. Our results indicate that HBK-10 had no agonistic properties, but it blocked the dopamine D_2_ receptor (Figure 3). In addition, its K_b_ was 3.1-fold lower than the K_b_ value for the reference antagonist, chlorpromazine (Table 5).

### 2.4. In Vivo Pharmacology

#### 2.4.1. HBK-10 Showed Antidepressant-like Activity in Mice after Acute Administration

**Figure 4:**
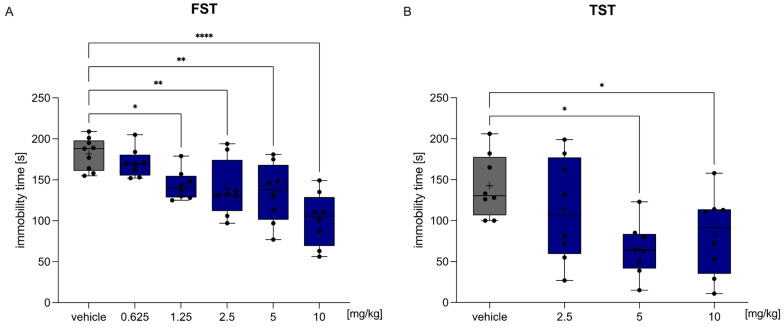
The effect of HBK-10 on the immobility of mice in the forced swim test (Panel (**A**)) and on the behavior of mice in the tail suspension test (Panel (**B**)). The tested compound or vehicle (1% Tween) were administered 30 min intraperitoneally (*ip*) before the test. The results are presented as box plots showing the following data: mean (‘+’), median (horizontal line), upper and lower quartile (the width of the box shows interquartile range), upper and lower extreme (whiskers). Statistical analysis: Shapiro–Wilk test for normality, Brown–Forsythe test for homogeneity of variance, and one-way ANOVA (Newman–Keuls post hoc); * *p* < 0.05, ** *p* < 0.01, **** *p* < 0.0001; *n* = 8–9 mice per group.

Since HBK-10 showed a high affinity for the 5-HT_1A_ receptor, we next investigated its potential antidepressant-like properties. We used two most common tests to evaluate antidepressant-like activity, i.e., the forced swim and tail suspension test [29]. In the forced swim test, the administration of HBK-10 at a dose range 1.25–10 mg/kg caused a significant reduction of immobility in the forced swim test by 21.1%, 23.5%, 26.6%, 44.2%, and 47.2%, respectively [F(5,43) = 9.042, *p* < 0.0001] (Figure 4A).

In the tail suspension test, the administration of HBK-10 at a doses of 5 and 10 mg/kg produced a significant reduction of immobility by 54.5% and 42%, respectively [F(3,28) = 4.156, *p* < 0.05] (Figure 4B).

#### 2.4.2. The Combined Administration of Sub-Effective Doses of HBK-10 and Fluoxetine Showed Antidepressant-like Effect

Since HBK-10 demonstrated antidepressant-like properties in two in vivo tests, we next investigated its possible mechanism of action. To do this, we performed experiments where we administered the compound jointly with antidepressants showing distinct mechanisms of action.

**Figure 5:**
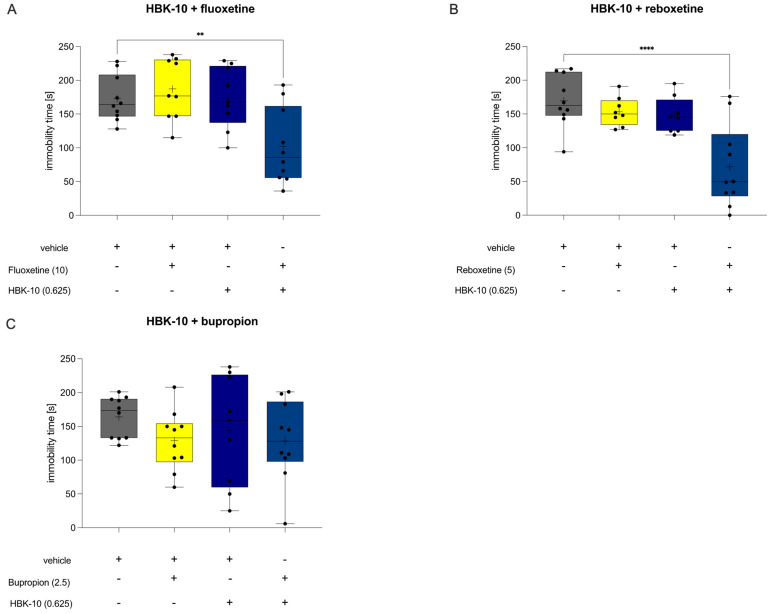
The effect of fluoxetine (Panel (**A**)), reboxetine (Panel (**B**)), and bupropion (Panel (**C**)) on antidepressant-like activity of HBK-10 in the forced swim test in mice. Studied compound and reference drugs: fluoxetine, reboxetine, and bupropion, were administered *ip* at sub-effective doses 30 min before the test. The results are presented as box plots showing the following data: mean (‘+’), median (horizontal line), upper and lower quartile (the width of the box shows interquartile range), upper and lower extreme (whiskers). Statistical analysis: Shapiro–Wilk test for normality, Brown–Forsythe test for homogeneity of variance, and two-way ANOVA (Newman–Keuls post hoc); ** *p* < 0.01, **** *p* < 0.0001; *n* = 8–10 mice per group.

We first utilized fluoxetine, a selective serotonin reuptake inhibitor. The two-way ANOVA demonstrated a significant effect of HBK-10 [F(1,34) = 7.896, *p* < 0.01], no effect of fluoxetine [F(1,34) = 3.728, ns], and a significant interaction between the treatments [F(1,34) = 8.441, *p* < 0.01]. The combined administration of fluoxetine (10 mg/kg) and HBK-10 (0.625 mg/kg) but not the compounds given alone significantly decreased the immobility by 40.9% in the forced swim test in mice (Figure 5A).

#### 2.4.3. The Combined Administration of Sub-Effective Doses of HBK-10 and Reboxetine Showed Antidepressant-like Effect

Next, we investigated the effect of the joint administration of HBK-10 and reboxetine, a noradrenaline reuptake inhibitor. The two-way ANOVA demonstrated that the overall effects of reboxetine [F(1,32) = 10.93, *p* < 0.01], HBK-10 [F(1,32) = 13.76, *p* < 0.001] and their interaction [F(1,32) = 4.664, *p* < 0.05] were found to be significant. The joint administration of reboxetine (5 mg/kg) and HBK-10 (0.625 mg/kg) but not the compounds given alone significantly decreased the immobility by 57.7% in the forced swim test in mice (Figure 5B).

#### 2.4.4. The Combined Administration of Sub-Effective Doses of HBK-10 and Bupropion Did Not Show Antidepressant-like Effect

Finally, we assessed the effect of a combined administration of sub-effective doses of HBK-10 and bupropion, a dopamine and noradrenaline reuptake inhibitor). The two-way ANOVA demonstrated no significant effect of bupropion [F(1,35) = 1.972, ns], HBK-10 [F(1,35) = 0.3143, ns], or their interaction [F(1,35) = 0.2959, ns]. The combined administration of bupropion (2.5 mg/kg) and HBK-10 (0.625 mg/kg) did not significantly decrease the immobility in the forced swim test in mice (Figure 5C).

#### 2.4.5. HBK-10 Did Not Show Antidepressant-like Activity after Pretreatment with pCPA

**Figure 6:**
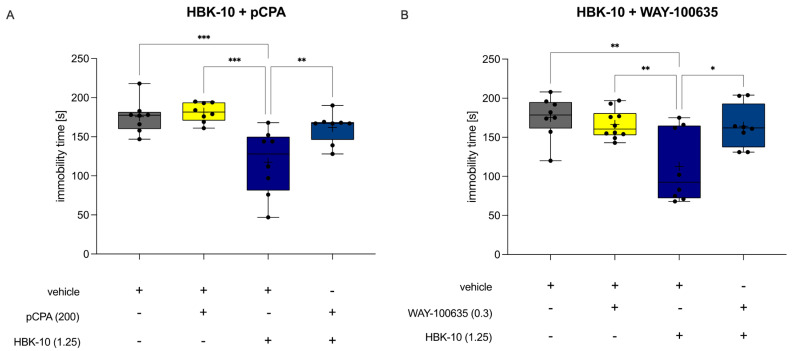
The effect of *p*-chlorophenylalanine (*p*CPA; Panel (**A**)), and WAY-100635 (Panel (**B**)) on antidepressant-like activity of HBK-10 in the forced swim test in mice. *p*CPA (200 mg/kg) was injected intraperitoneally (*ip*) once daily for three consecutive days. 24 h after the last injection and 30 min before the test mice received *ip* either studied compound or vehicle (1% Tween). WAY-100635 (0.1 mg/kg) was administered subcutaneously (*sc*) 15 min before tested compound, then 30 min before the test mice received *ip* either studied compound or vehicle (1% Tween). The results are presented as box plots showing the following data: mean (‘+’), median (horizontal line), upper and lower quartile (the width of the box shows interquartile range), upper and lower extreme (whiskers). Statistical analysis: Shapiro–Wilk test for normality, Brown–Forsythe test for homogeneity of variance, and two-way ANOVA (Newman–Keuls post hoc); * *p* < 0.05, ** *p* < 0.01, *** *p* < 0.001; *n* = 8–10 mice per group.

Since experiments with antidepressants demonstrated that serotonergic system plays an important role in the mechanism of action of HBK-10, we next performed another set of experiments with *p*CPA, a tryptophan hydroxylase inhibitor, which diminishes brain serotonin levels [30]. The two-way ANOVA demonstrated a significant effect of HBK-10 [F(1,28) = 17.67, *p* < 0.0001], *p*CPA [F(1,28) = 7.369, *p* < 0.05] and their interaction [F(1,28) = 4.376, *p* < 0.05]. HBK-10 (1.25 mg/kg) significantly reduced immobility of mice by 28.1% (Figure 4A). Pretreatment with *p*CPA (200 mg/kg) had no effect on the immobility but it significantly abolished the effect produced by HBK-10 (Figure 6A).

#### 2.4.6. HBK-10 Did Not Show Antidepressant-like Activity after Pretreatment with WAY-100635

Given HBK-10 is a serotonin 5-HT_1A_ receptor ligand, we investigated whether this receptor participates in the compound’s mechanism of action. To do that, we performed experiments with WAY-100635, an antagonist of the 5-HT_1A_ receptor [31]. The two-way ANOVA demonstrated no significant effect of WAY-100635 [F(1,29) = 3.648, ns], a significant effect of HBK-10 [F(1,29) = 8.604, *p* < 0.01], and a significant interaction [F(1,29) = 7.403, *p* < 0.05]. The administration of HBK-10 (1.25 mg/kg) significantly reduced immobility of mice by 35.7% (Figure 4B). Pretreatment with WAY-100635 (0.1 mg/kg) had no effect on the immobility but it significantly abolished the effect produced by HBK-10 (Figure 6B).

#### 2.4.7. HBK-10 Showed Antidepressant-like Activity in the Forced Swim Test in the Corticosterone-Treated Mice after a Chronic Administration

**Figure 7:**
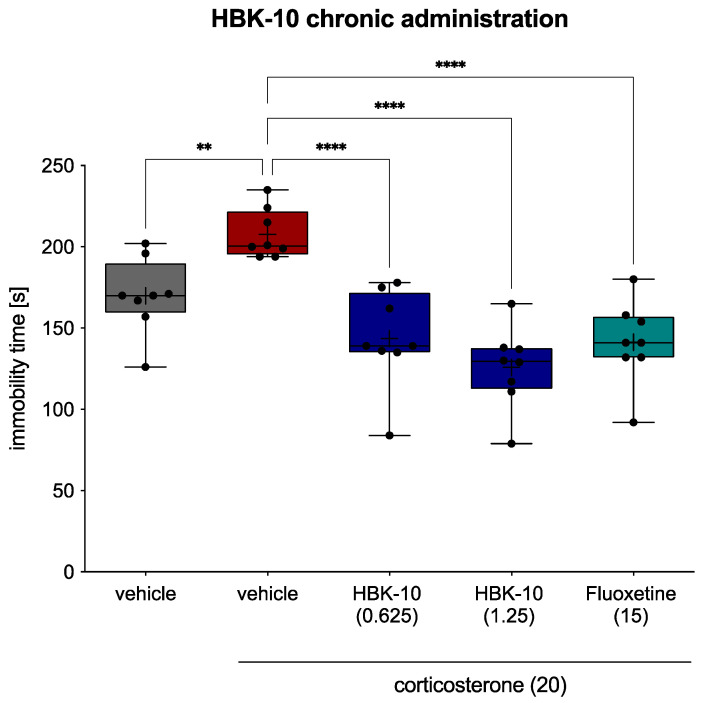
The effect of HBK-10 and fluoxetine on the immobility in the forced swim test after a chronic administration in corticosterone-treated mice. After 3 weeks of either corticosterone or vehicle injections *sc*, mice were administered *ip* with HBK-10 (0.625 or 1.25 mg/kg), fluoxetine (15 mg/kg), or vehicle (1% Tween) for next 3 weeks, 30 min before the corticosterone or vehicle injections. 24 h after the last compounds’ administration, the forced swim test was performed. The results are presented as box plots showing the following data: mean (‘+’), median (horizontal line), upper and lower quartile (the width of the box shows interquartile range), upper and lower extreme (whiskers). Statistical analysis: Shapiro–Wilk test for normality, Brown–Forsythe test for homogeneity of variance, and one-way ANOVA (Newman–Keuls post hoc); ** *p* < 0.01, **** *p* < 0.0001; *n* = 8 mice per group.

Since tests such as the forced swim and tail suspension provide only a behavioral measure designed to assess the effect of pharmacological manipulation, we next decided to evaluate the antidepressant-like potential of HBK-10 in depression model. We used a corticosterone-induced model of depression in mice [32]. Our experiments demonstrated that the corticosterone-treated mice showed increased immobility (by 22.3%) compared to the vehicle-treated control [F(4,35) = 14.13, *p* < 0.0001] (Figure 7). Both HBK-10 at doses 0.625 and 1.25 as well as fluoxetine reversed the corticosterone-induced changes by decreasing the measured parameter by 30.9%, 39.5%, and 32%, respectively (Figure 7).

#### 2.4.8. HBK-10 Did Not Alter the Locomotor Activity in the Corticosterone-Treated Mice after a Chronic Administration

Since psychostimulants can show false positive results in the forced swim test [33], we assessed the influence of the compound on locomotor activity in corticosterone-treated mice. None of the treatments influenced the number of crossings of photobeams over the 4-min testing period in mice [F (4,35) = 0.9438, ns] (Table 6).

#### 2.4.9. HBK-10 Decreases Locomotor Activity in Mice at Higher Doses

We next investigated whether HBK-10 influences locomotor activity of mice at higher doses. Our experiments showed that HBK-10, given alone, at doses 40 and 60 mg/kg decreased locomotor in mice [F(4,38) = 18.91, *p* < 0.0001] (Table 7). Neither concomitant administration of HBK-10 with antidepressant drugs nor *p*CPA or WAY-100635 influenced the mice’ locomotor activity (data not shown).

#### 2.4.10. HBK-10 Influenced Motor Coordination in Mice at Highest Dose Tested

Some central-acting drugs may negatively influence brain function [34,35,36]. Thus, we next investigated whether HBK-10 affects motor coordination in mice. In the rotarod test HBK-10 [H(5,40) = 21.51, *p* < 0.001] affected the motor coordination at the dose 60 mg/kg, but has no influence on measured parameter at the doses active in the forced swim and tail suspension tests (Table 8).

In the chimney test HBK-10 [H(6,60) = 18.83, *p* < 0.01] impaired the motor coordination at the dose 60 mg/kg, but had no effect on measured parameter after administration at the doses active in the forced swim and tail suspension tests (Table 8).

## 3. Discussion

In this study, we demonstrated the synthesis and antidepressant-like properties of HBK-10, a novel 2-methoxyphenyl derivative. The compound presented a high affinity for the 5-HT_1A_ receptors and relatively high for D_2_ receptors. In cAMP (5-HT_1A_) and calcium mobilization (D_2_) assays, HBK-10 acted as an antagonist of these receptors. The tested compound elicited antidepressant-like properties not only in naïve but also in corticosterone-treated mice (model of depression). Its antidepressant-like effect was most likely related to the influence on serotonergic and noradrenergic neurotransmission, particularly interaction with the 5-HT_1A_ receptor. Notably, the studied 2-metoxyphenylpiperazine derivative did not impair motor function in mice at antidepressant-like doses.

First, after synthesizing HBK-10, we assessed whether the compound is suitable for in vivo studies. In order to do that, we investigated its metabolic stability and potential hepatotoxic properties. Our experiments demonstrated that HBK-10 did not modify HepG2 cells viability, suggesting that it is not hepatotoxic at concentrations below 25 µM. Next, we predicted the level of the first-pass metabolism of the tested compound in mice. We compared the obtained results to the literature data for buspirone, an arylpiperazine drug subjected to the first-pass effect in humans [38]. As the recovery percentage of buspirone after 30 min of incubation with MLMs was lower than 1%, the drug showed a rapid microsomal metabolism in mice [38]. However, for HBK-10, the value of recovery percent of parent compound after the same time was more than 100-fold higher, suggesting that the compound is more metabolically stable than buspirone. Next, we assessed the half-life and the intrinsic clearance in vitro of HBK-10 and compared them with literature data for aripiprazole and imipramine. HBK-10 showed higher stability than a tricyclic antidepressant and lower than aripiprazole. Thus, our analysis showed that HBK-10 is a medium-clearance compound suitable for in vivo studies [39].

In the following step, we investigated the in vitro profile of the studied compound. Numerous studies showed that 2-methoxyphenyl derivatives are ligands of serotonin and dopamine receptors [18,23]. Thus, we first assessed the compound’s affinity towards serotonin 5-HT_1A_, 5-HT_2A_, 5-HT_6_, 5-HT_7_, and dopamine D_2_ receptors. HBK-10 exhibited a high affinity for serotonergic 5-HT_1A_ and a relatively high affinity towards dopaminergic D_2_ receptors. Its affinity for the rest of the tested biological targets was either moderate or very low. Given the high affinity of HBK-10 for 5-HT_1A_ and D_2_ receptors, we next determined the type of interaction with these receptors by performing functional studies. The tested compound showed antagonistic properties at both 5-HT_1A_ and D_2_ receptors. Our results agree with our previous studies on aroxyalkilo-*N*-(2-methoxyphenyl)piperazine derivatives, which demonstrated high affinity for 5-HT_1A_ and D_2_ receptors, as well as antagonistic properties at 5-HT_1A_ and D_2_ receptors in cAMP inhibition or Ca^2+^ mobilization in vitro functional assays [15,18,19,22,40].

Given the significant role of the 5-HT_1A_ receptor in depression and the high affinity of HBK-10 for this receptor, we decided to investigate the antidepressant-like properties of HBK-10 using two standard behavioral tests in mice—forced swim and tail suspension tests. The compound showed antidepressant-like activity in both tests. However, the results indicate that HBK-10 was active at a wider dose range in the forced swim test than in the tail suspension test. These differences may be explained by the fact that the tail suspension test is more sensitive to the sedative properties of compounds, especially targeting 5-HT_1A_ receptors [41]. Moreover, the divergent results might be due to the differences in the procedures themselves, i.e., test duration, environment, risk of hypothermia in the forced swim test. In the forced swim test, HBK-10 showed antidepressant-like activity at a 2-fold lower dose than escitalopram or 8-fold lower than reboxetine [19,42]. HBK-10 did not influence locomotor activity of mice at antidepressant-like doses. Therefore, the results of the above behavioral tests cannot be attributed to the psychostimulant properties of the compound.

To find a possible mechanism of the antidepressant-like effect of HBK-10, we performed another set of behavioral experiments. Since HBK-10 possessed a high affinity towards serotonergic and dopaminergic receptors and these receptors play a significant role in depression and antidepressant activity, we investigated the effects of simultaneous administration of sub-effective doses of tested compound and antidepressants with different pharmacological profiles. We used fluoxetine, a selective serotonin reuptake inhibitor, reboxetine, a selective noradrenaline reuptake inhibitor, and bupropion, dopamine, and noradrenaline reuptake inhibitor. Interaction studies with conventional antidepressants help determine which system is involved in the antidepressant-like effect of the studied compound [43]. As we observed an additive effect after joint administration of HBK-10 with fluoxetine or reboxetine (but not bupropion), the antidepressant-like effect of HBK-10 might be mediated via serotonergic and noradrenergic systems. Our results cannot be linked to a psychostimulant combination of these compounds, since neither HBK-10 nor its combination with fluoxetine or reboxetine influenced locomotor activity of animals.

Since studies with sub-effective doses of antidepressants showed that the serotoninergic system plays a part in the HBK-10 mechanism of action, we next performed another experiment to prove the significant role of this system in the antidepressant-like effect of the compound. To do that, we pretreated mice with *p*CPA, a selective inhibitor of tryptophan hydroxylase, which diminishes brain serotonin levels [30]. According to Imaizumi and colleagues, a 3-day treatment with *p*CPA causes a significant reduction of serotonin level in the cortical regions by around 30% and in midbrain by 24% [30]. Our results showed that the administration of *p*CPA abolished the behavioral effect of HBK-10 in the forced swim test. This suggests that to exert an antidepressant-like effect, HBK-10 requires the availability of serotonin in the synaptic cleft. As expected, the results are in agreement with our previous studies on 2-metoxyphenypiperazine derivatives [19,20,21], as well as studies of other researchers [44,45,46,47,48].

Since the serotonergic system plays a vital role in the HBK-10 mechanism of action and the compound showed a high affinity for the 5-HT_1A_ receptor, we investigated whether the receptor contributes to the compound’s antidepressant-like activity. To test the role of the 5-HT_1A_ receptor in the antidepressant-like effect of HBK-10, we pretreated mice with WAY-100635, a 5-HT_1A_ receptor antagonist. Interestingly, WAY-100635 abolished the antidepressant-like activity of HBK-10. We suspect that the antidepressant-like effect of HBK-10 may be due to the blockade of the presynaptic 5-HT_1A_ autoreceptors, which in consequence increases the serotonin levels, which in turn stimulate postsynaptic 5-HT_1A_ receptors. We obtained similar results for several previously synthesized compounds with 2-methoxyphenylpiperazine moiety [49]. However, our theory needs further confirmation. HBK-10 might also be a functionally selective 5-HT_1A_ receptor agonist, which shows antagonistic properties at one pathway, while agonistic properties at another signaling pathway coupled with the 5-HT_1A_ receptor. As mentioned earlier, studies demonstrated that the 5-HT_1A_ receptor couples to a broad array of signaling pathways [8]. Here we tested the influence of HBK-10 on only one, canonical signaling pathway, i.e., cAMP inhibition. Our former experiments with other 2-methoxyphenylopiperazine derivatives showed that these compounds antagonized cAMP inhibition and Ca^2+^ mobilization after binding to the 5-HT_1A_ receptor [19,22,40,50]. On the other hand, the same compounds demonstrated agonistic properties at the β-arrestin pathway and increased the phosphorylation of ERK1/2 [40,50]. Interestingly, the phosphorylation of ERK1/2 is decreased in post mortem brain tissues of suicide victims [51] and is crucial for the antidepressant-like effect of a rapid-acting antidepressant—ketamine [52]. Therefore, HBK-10, like other 2-methoxyphenylpiperazine derivatives that we have synthesized, might activate the β-arrestin pathway and increase the phosphorylation of ERK1/2 while blocking other signaling pathways. This in turn, might explain why WAY-100635 blocked the antidepressant-like effect of HBK-10 in vivo. Nevertheless, this hypothesis requires further in vitro studies. Finally, the antidepressant-like effect of HBK-10 may be a result of the interaction with different molecular targets. However, based on the above results, we can assume that the behavioral effect of HBK-10 depends on the serotonergic neurotransmission and is specifically mediated via 5-HT_1A_ receptors.

Tests such as the forced swim test provide only an endpoint—a behavioral measure (read-out) designed to assess the effect of pharmacological manipulation. However, depression models can reproduce aspects of human pathology and provide a certain degree of predictive validity [7,53]. Thus, to increase the translational potential of our study, we next investigated the antidepressant-like effect of HBK-10 in a mouse depression model induced by chronic corticosterone administration. The corticosterone-induced model of depression is widely used in preclinical experiments (reviewed in [54]). The repeated administration of this glucocorticoid hormone induces behavioral and biochemical changes in animals similar to those observed in depression [55,56,57,58]. Chronic antidepressant treatment reverses these changes [52].

To assess the antidepressant-like effect of HBK-10 in the corticosterone-induced depression model, we chose two doses of the tested compound—the lowest active and the first inactive in the forced swim test in naïve mice, i.e., 1.25 mg/kg and 0.625 mg/kg. We showed that corticosterone-treated mice were more immobile in the forced swim test compared to vehicle-treated controls, which suggests the development of depressive-like behaviors. A chronic administration of HBK-10 (0.625 and 1.25 mg/kg) as well as the reference compound, fluoxetine, reversed the corticosterone-induced changes. The behavioral effect of HBK-10 was similar to fluoxetine, although it was achieved at a 24-fold lower dose than the reference compound. Interestingly, in naïve animals, the dose of 0.625 mg/kg was inactive after a single administration, but a chronic administration (3 weeks) of the same dose was enough to reverse the corticosterone-induced increase in mice immobility. Since none of the treatments affected the locomotor activity of mice, the observed results were specific to antidepressant-like effect.

Finally, we aimed to assess the preliminary safety profile of HBK-10, i.e., its influence on locomotor activity and motor coordination in mice. We demonstrated that HBK-10 displays sedative properties; however, the ED_50_ value for sedative effect was approximately 26-fold higher than the lowest antidepressant-like dose. Thus, there is a very low likelihood of sedation when administering HBK-10 at antidepressant-like doses.

Many centrally acting drugs, including antidepressants, impair motor coordination [34,35,59]. Thus, we also assessed whether HBK-10 affects the animals’ motor performance in two assays: rotarod and chimney tests. Similar to the effect on the locomotor activity, HBK-10 impaired motor abilities at higher doses than those needed for antidepressant-like activity—TD_50_ values were approximately 34-fold higher than the lowest antidepressant-like dose. This suggests that it is unlikely that HBK-10 will cause motor coordination impairments at antidepressant-like doses.

Interestingly, the pharmacological profile of HBK-10 in the above behavioral tests resembles the profile of HBK-15, a compound showing a rapid antidepressant-like effect in rodent models of depression, as well as additional pharmacological properties, such as anxiolytic-like or procognitive effects [18,19,20,21,22,60,61,62]. Both compounds show high affinity for the 5-HT_1A_ and D_2_ receptors, a wider active dose range in the forced swim test than the tail suspension test, and antidepressant-like effect at lower doses than the reference antidepressants. The advantage of HBK-10 over HBK-15 is that it showed antidepressant-like effect at a 2-fold lower dose than HBK-15 in the corticosterone-induced model of depression [21], which may indicate its higher pharmacological activity. Given the similarities between the compounds, HBK-10 is worthy of further investigation.

Limitations to this study include assessing the antidepressant-like activity of HBK-10 after only chronic administration and not investigating its effect on neurobiological changes in the depression model. Evaluating the ability of a single administration of HBK-10 to reverse the behavioral and neurobiological changes induced by chronic corticosterone injection would give insight into whether the compound, similar to HBK-15, has the potential to be a rapid-acting antidepressant. In addition, investigating the HBK-10 influence on neurobiological alterations in the depression model would help explain the compound’s mechanism of action. Moreover, as a 2-methoxyphenylpiperazine derivative, HBK-10 might target adrenergic receptors, which might play a role in its pharmacological activity, but can also cause unwanted effects, including lowering blood pressure. Thus, this issue requires further investigation. Finally, the tested compound is a racemate, so it is possible that its antidepressant-like activity is mediated by only one enantiomer. Therefore, our future research will focus on the synthesis of enantiomers and their extensive pharmacological characterization.

## 4. Materials and Methods

### 4.1. Chemistry

All commercially available chemicals and reagents were used without any further purification. Melting points of compounds were measured on Büchi SMP-560 apparatus (Büchi Labortechnik AG, Flawil, Switzerland) and were given as uncorrected. NMR spectra were recorded on a JEOL ECZ-500R spectrometer equipped with a JEOL Royal Probe HFX (JEOL USA Inc., Peabody, MA, USA) at 500 MHz for ^1^H and at 126 MHz for ^13^C; with solvent as the internal standard. The UPLC–MS system equipped with a Waters ACQUITY® UPLC® (Waters Corporation, Milford, MA, USA) and a Waters TQD mass spectrometer (electrospray ionization (ESI) mode tandem quadrupole) was used for the analysis. The investigated compounds were analyzed on a Acquity UPLC BEH (bridged ethyl hybrid) C18 column (2.1 × 100 mm, 1.7 µm), comprising an Acquity UPLC BEH C18 VanGuard precolumn (2.1 × 5 mm, 1.7 µm). The purity of compounds was determined by UPLC-MS and was above 98%.

#### Synthesis

The following substrates and their methods of synthesis (Scheme 1) were already described: 3-(2,6-dimethylphenoxy)propan-1-ol (**1**), 2-(3-bromopropoxy)-1,3-dimethylbenzene (**2**) [14]; 2-(1-hydroxybutan-2-yl)isoindoline-1,3-dione (**3**), 2-(1-bromobutan-2-yl)isoindoline-1,3-dione (**4a**) [24].

2-(1,3-dioxoisoindolin-2-yl)butyl 4-methylbenzenesulfonate (**4b**)

A mixture of **3** (0.1 mol) and *p*-toulenesulfochloride (0.1 mol) in toluene (100 mL) was heated under reflux in the presence of pyridine (0.11 mol) for 2 h. After distillation of solvent the product was washed with water and the crystallized from toluene/n-hexane (3/1) to give solid **4b**.

2-(1,3-dioxoisoindolin-2-yl)butyl 4-methylbenzenesulfonate **(4b)**

White solid, yield 67%. M.p. 112–114 °C. M 373.42. ^1^H NMR (500 MHz, DMSO-*d*6) δ ppm 7.79–7.86 (m, 2 H, Ar-H), 7.73–7.79 (m, 2 H, Ar-H), 7.53 (d, *J* = 8.31 Hz, 2 H, Ar-H), 7.17 (d, *J* = 7.73 Hz, 2 H, Ar-H), 4.46 (m, 1 H, O-CHH), 4.26–4.31(m, 1 H, O-CHH), 4.21 (tt, *J* = 9.95, 4.80 Hz, 1 H, N-CH), 2.22–2.26 (s, 3 H, CH_3_-Ar), 1.72–1.85 (m, 1 H, CH_3_-CHH), 1.60–1.70 (m, 1 H, CH_3_-CHH), 0.71 (t, *J* = 7.45 Hz, 3 H, CH_3_-CH_2_).

ESI-MS Calcd. for C_19_H_20_NO_5_S [M + H]^+^ 374.106; Found 374.184.

2-(1-(4-(2-methoxyphenyl)piperazin-1-yl)butan-2-yl)isoindoline-1,3-dione **(5)**

To a mixture of 2-methoxyphenylpiperazine (0.05 mol) and K_2_CO_3_ (0.027 mol) in 50 mL of 2-methoxyethanol compound **4b** (0.051 mol) was added and the reaction was refluxed for 5 h. The inorganic salts were filtered off and the filtrate was evaporated to dry residue. After addition of *n*-heptane, the mixture was refluxed and cooled to rt. Recrystallization from toluene/*n*-heptane (1/2) yielded **5**.

2-(1-(4-(2-methoxyphenyl)piperazin-1-yl)butan-2-yl)isoindoline-1,3-dione **(5)**

White solid, yield 38%. M.p. 172–173 °C M 393.49. 1H NMR (500 MHz, CHLOROFORM-*d*) δ ppm 7.81 (dd, *J* = 5.44, 2.86 Hz, 2 H, Ar-H), 7.65–7.72 (m, 2 H, Ar-H), 6.88–6.97 (m, 1 H, Ar-H), 6.77–6.86 (m, 3 H, Ar-H), 4.35 (tt, *J* = 10.27, 5.05 Hz, 1 H, N-CH), 3.81 (s, 3 H, CH_3_-O), 2.46–3.18 (m, 10 H, N-CH_2_, pip-CH_2_), 2.00–2.12 (m, 1 H, CH_3_-CH_2_), 1.70–1.83 (m, 1 H, CH_3_-CH_2_), 0.91 (t, *J* = 7.45 Hz, 3 H, CH_3_-CH_2_). ESI-MS Calcd. for C_23_H_28_N_3_O_3_ [M + H]^+^ 394.213; Found 394.261.

1-(4-(2-methoxyphenyl)piperazin-1-yl)butan-2-amine (**6**)

To the solution of **5** (0.015 mol) in ethanol (50 mL) 4 mL of 98% hydrazine was added and heated in water bath for 1 h. The white precipitate of phtalhydrazide was filtered off, the filtrate was cooled and spiked with 2 mL of conc. hydrochloric acid. The mixture was heated for the next 1 h and again the phtalhydrazide was filtered off and washed with water. The combined filtrates were concentrated in vacuo, alkalized with NaOH (15%) and the oil product was extracted to dichloromethane (3 times, 20 mL). Combined organic layers were dried over Na_2_SO_4_ and evaporated in vacuo to yield the compound **6**, as oily reside, which was subjected to the next step without further purification.

*N*-(3-(2,6-dimethylphenoxy)propyl)-1-(4-(2-methoxyphenyl)piperazin-1-yl)butan-2-amine dihydrochloride (HBK-10)

The title compound was synthesized as per the previously reported method [14,16]. A mixture of crude **6** (0.01mol), 2-(3-bromopropoxy)-1,3-dimethylbenzene (**2**) (0.011 mol) and K_2_CO_3_ (0.007 mol) in toluene (50 mL) was refluxed for 8h. The inorganic precipitate was filtered off, washed with hot toluene, and the combined filtrates were concentrated under vacuo to dry residue. The water (40 mL) was added to the residue and the mixture was acidified with HCl (10%). After activated carbon was added the mixture was heated and filtered. Then the solution was cooled to the rt, and it was alkalized with NaOH (15%) to get semi-solid basis of HBK-10. Further it was extracted to dichloromethane (three times, 20 mL), after drying over anhydrous Na_2_SO_4_ and evaporated in vacuo to dry residue, that was heated in acetone/ethanol with activated carbon. After filtering off, the solution was cooled and saturated with gaseous HCl to get the white solid, that was recrystallized from acetone/ethanol (5/1) to yield HBK-10.

*N*-(3-(2,6-dimethylphenoxy)propyl)-1-(4-(2-methoxyphenyl)piperazin-1-yl)butan-2-amine dihydrochloride (HBK-10)

White solid, yield 64%. M.p. 224–226 °C. M 498.23. 1H NMR (500 MHz, DMSO-d_6_) δ ppm 10.90–11.26 (m, 1 H, NH_4_^+^), 9.31–9.95 (m, 2 H, NH_4_^+^), 6.85–7.02 (m, 7 H, Ar-H), 3.80 (t, *J* = 6.30 Hz, 2 H, CH_2_-O), 3.76 (s, 3 H, CH_3_-O), 3.51–3.61 (m, 4 H, 2x Pip-CH_2_), 3.08–3.24 (m, 7 H, 2x Pip-CH_2_, CH-CH_2_, CH_2_-N-Pip), 2.16–2.22 (m, 10 H, 2x CH_3_-Ar, CH_2_-CH_2_-CH_2_), 1.82–1.95 (m, 1 H, CH_3_-CHH), 1.59–1.69 (m, 1 H, CH_3_-CHH), 0.97 (t, *J* = 7.30 Hz, 3 H, CH_3_-CH_2_). ^13^C NMR (126 MHz, DMSO-d6) δ ppm 155.77, 152.38, 139.77, 130.87, 129.28, 124.36, 124.12, 121.39, 118.82, 112.46, 69.32, 56.17, 55.91, 54.13, 53.48, 51.27, 47.45, 42.26, 40.62, 40.45, 40.28, 40.11, 27.38, 21.72, 16.57, 9.97. ESI-MS Calcd. for C_26_H_40_N_3_O_2_ [M + H]^+^ 426.312; Found 426.419.

### 4.2. In Vitro Studies

#### 4.2.1. Cytotoxicity

To investigate the preliminary hepatic safety of HBK-10 at the concentration used in metabolic stability study, a human hepatocellular carcinoma cell line—HepG2 (HB-8065TM, ATCC, Manassas, VA, USA) was used in the study. HepG2 cells were cultured in standard conditions (37 °C, 5% CO_2_, 95% humidity) in appropriate culture medium (according to manufacturer procedure), supplemented with 10% fetal bovine serum (FBS, Gibco, Thermo Fisher Scientific, Waltham, MA, USA) and antibiotics (1% streptomycin/penicillin mixture; Sigma-Aldrich, Steinheim, Germany). Cells were seeded in 96-well plates at density of 1 × 10^4^ per well. After 24 h cells were treated with two doses of HBK-10 (5 and 25 µM, solvent—PBS) and incubated for additional 24 h. Following the incubation cells viability was measured with MTT assay. For this purpose, MTT reagent (3-(4,5-dimethylthiazol-2-yl)-2,5-diphenyltetrazolium bromide) (Sigma Aldrich, Steinheim, Germany) was added to each well at the concentration 0.5 mg/mL. After 4 h incubation at 37 °C, the medium was aspirated, and formazan crystals produced in the cells were dissolved in DMSO. Then the absorbance of solution was determined at 570 nm (A570) on plate reader (Spectra iD3 Max, Molecular Devices; San Jose, CA, USA). Viability (% of control) was determined by dividing A570 of experimental wells by of A570 of control wells × 100%.

#### 4.2.2. Metabolic Stability

The assay was performed with mouse liver microsomes from male CD-1. Incubations were performed in duplicates as follows: microsomes in 0.1 M potassium phosphate buffer pH 7.4 (0.8 mg/mL microsomal protein), tested compound (final concentration 20 μM, cosolvent methanol, final concentration 0.38%) were preincubated at 37 °C for 15 min. Metabolic reactions were initiated by the addition of the NADPH regenerating system, containing NADP, glucose-6-phosphate, and glucose-6-phosphate dehydrogenase. Mixtures were incubated at 37 °C for various time periods (15, 30, 60 min). Enzymatic reactions were quenched by perchloric acid followed by addition of internal standard (pentoxifylline). Negative controls were performed without NADPH-regenerating system. After centrifugation, samples were analyzed by LC/MS (UPLC/MS, Waters Corporation, Milford, MA, USA). In vitro half time (t_1/2_) and intrinsic clearance (Cl_int_) of test compound in liver microsomes were determined according to literature procedures [26,63].

#### 4.2.3. Radioligand Binding Assays

Radioligand binding was performed on membranes from CHO-K1 cells stably transfected with the human 5-HT_1A_, 5-HT_2A_, 5-HT_6_, or 5-HT_7_ receptors. The affinity for the serotonin transporters was performed using rat cortex tissue. Binding experiments were conducted in 96-well microplates, and the reaction mix included the solution of the test compound, radioligand, and diluted membranes or the tissue suspension.

Specific assay conditions are shown in Table 9. The reaction was terminated by rapid filtration through GF/B or GF/C filter mate presoaked with 0.3% polyethyleneimine for 30 min. Ten rapid washes with 200 µl 50 mM Tris buffer (4 °C, pH 7.4) were performed using an automated harvester system Harvester-96 MACH III FM (Tomtec). The filter mates were dried at 37 °C in a forced air fan incubator, and then solid scintillator MeltiLex was melted on filter mates at 90 °C for 5 min. Radioactivity was counted in the MicroBeta2 scintillation counter (PerkinElmer) at approximately 30% efficiency. The concentration of the analyzed compounds ranged from 10^−10^ to 10^−5^ M. The inhibitory constant (*K_i_*) was estimated. A single assay was performed with each compound concentrations in duplicate, and the whole assay was repeated in a three independent experiments.

#### 4.2.4. Functional Assays for 5-HT_1A_ Receptor

Tested and reference compounds were dissolved in dimethyl sulfoxide (DMSO) at a concentration of 10 mM. Serial dilutions were prepared in 96-well microplate in assay buffer, and 8 to 10 concentrations were tested. For the 5-HT_1A_, adenylyl cyclase activity was monitored using cryopreserved CHO-K1 cells with expression of the human serotonin 5-HT_1A_ receptor. A functional assay based on cells with expression of the human hydroxytryptamine (serotonin) receptor 1A was performed. Thawed cells were resuspended in stimulation buffer (HBSS, 5 mM HEPES, 0.5 IBMX, and 0.1% BSA at pH 7.4) at 2 × 10^5^ cells/mL. The 10 µL of cell suspension was added to tested compounds with 10 µM forskolin. Samples were loaded onto a white opaque half area 96-well microplate. The antagonist response experiment was performed with 30 nM serotonin as the reference agonist. The agonist and antagonist were added simultaneously. Cell stimulation was performed for 40 min at room temperature. After incubation, cAMP measurements were performed with homogeneous TR-FRET immunoassay using the LANCE Ultra cAMP kit (PerkinElmer, Waltham, MA, USA). Ten microliters of EucAMP Tracer Working Solution and 10 µl of ULight-anti-cAMP Tracer Working Solution were added, mixed, and incubated for 1 h. The TR-FRET signal was read on an EnVision microplate reader (PerkinElmer, USA). IC50 and EC50 were determined by nonlinear regression analysis using GraphPad Prism 6.0 software.

#### 4.2.5. Functional Assays for D_2_ Receptor

Tested and reference compounds were dissolved in dimethyl sulfoxide (DMSO) at a concentration of 1 mM. Serial dilutions were prepared in 96-well microplate in assay buffer, and 8-to-10 concentrations were tested. A cellular aequorin-based functional assay was performed with recombinant CHO-K1 cells expressing mitochondrially targeted aequorin, human GPCR, and the promiscuous G protein Gαqi/5 for D_2_ receptor. After thawing, cells were transferred to assay buffer (DMEM/HAM’s F12 with 0.1% protease-free BSA) and centrifuged. The cell pellet was resuspended in assay buffer, and coelenterazine h was added at final concentrations of 5 µM. The cells suspension was incubated at 16 °C, protected from light with constant agitation for 16 h, and then diluted with assay buffer to the concentration of 100,000 cells/mL. After 1 h of incubation, 50 µL of the cell’s suspension was dispensed using automatic injectors built into the radiometric and luminescence plate counter MicroBeta2 LumiJET (PerkinElmer, USA) into white opaque 96-well microplates preloaded with test compounds. Immediate light emission generated following calcium mobilization was recorded for 30 s. In antagonist mode, after 30 min of incubation, the reference agonist was added to the above assay mix and light emission was recorded again. The final concentration of the reference agonist was equal to EC80 (30 nM apomorphine).

### 4.3. In Vivo Studies

#### 4.3.1. Animals

Adult male Albino-Swiss mice (CD-1, 8 weeks old, 18–21g: Jagiellonian University Medical College, Krakow, Poland) were used in all experiments. Unless stated otherwise, the animals were kept in groups of 10 mice in standard cages (37 cm × 21 cm × 15 cm) at a room temperature (22 ± 2 °C), on a 12 h light/dark cycle (lights on 7:00) with ad libitum access to food and water. Mice were used only once in each test. Behavioral experiments were performed between 8 am and 4 pm and evaluated by a trained observer blind to the treatments. Mice were handled for 1 week before starting the experimental procedures. Animals were randomly allocated to the treatment using a computer-generated sequence, and researchers making measurements on the animals or analyzing the results were blind to the allocation. Moreover, experimental groups were distributed across multiple cages, and the location of the mouse cages in the room was changed following each day.

#### 4.3.2. Drugs

HBK-10, WAY-100635 (Sigma, Darmstadt, Germany), *p*-chlorophenylalanine (*p*CPA; Sigma, Darmstadt, Germany), fluoxetine (Sigma, Darmstadt, Germany), reboxetine (Sigma, Darmstadt, Germany) and bupropion (Sigma, Darmstadt, Germany), were suspended in 1% Tween, and administered *ip* at a volume of 10 mL/kg 30 min before each behavioral test. The control groups received saline *ip* 30 min prior to testing. The doses of fluoxetine, reboxetine and bupropion as well as WAY-100635 and *p*CPA were based on our previous studies with 2-methoxyphenylpiperazine derivatives [18,42,64].

#### 4.3.3. Forced Swim Test

The experiment was performed on mice according to the method described by Porsolt et al. and as previously described [60,65]. Mice were placed individually in glass cylinders (height 25 cm, diameter 10 cm) filled with water at 24 ± 1 °C to a depth of 10 cm and left there for 6 min. Following a 2 min habituation period, total time spent immobile was recorded during the next 4 min. The animal was considered as immobile when it remained floating passively in the water, making only small movements to keep its head above the water. The experiments were video-recorded and scored using elevenmaze.com software by a trained observer blind to the treatments.

##### Serotonin Synthesis Blockade

To assess the involvement of a serotonergic system in the antidepressant-like activity of tested compounds in the forced swim test, the mice were pretreated with *p*CPA (tryptophan hydroxylase inhibitor) according to the method previously described [64]. We injected mice with either *p*CPA at a dose of 200 mg/kg or 1% Tween for three consecutive days. Twenty-four hours after the last *p*CPA administration, we injected mice with 1% Tween *ip* or tested compound. We performed the forced swim test 30 min after the administration.

##### The Blockade of 5-HT_1A_ Receptors

The putative involvement of 5-HT_1A_ receptors in the antidepressant-like effect of tested compound was studied using WAY-100635 (a selective 5-HT_1A_ receptor antagonist). WAY-100635 at a dose of 0.1 mg/kg was administered *sc* 15 min prior to the injection of HBK-10. After 30 min the forced swim test was performed. The behaviorally ineffective dose of WAY-100635 used in the experiments was based on our previous experiments [64].

#### 4.3.4. Tail Suspension Test

The experiment was carried out in mice according to the method described by Steru et al. and as previously described [66,67]. The mice were suspended by their tails using a medical adhesive tape at the height of 50 cm above a flat surface, in such a position that they cannot escape or hold on to nearby surfaces. The total time of immobility was measured during the 6-min test period. Immobility was defined as the animal hanging passively without limb movement. The experiments were video-recorded and scored using elevenmaze.com software by a trained observer blind to the treatments.

#### 4.3.5. Spontaneous Locomotor Activity in Mice

The locomotor activity of mice was measured as previously described [18]. We used photoresistor actometers (Ugo Basile, Italy) connected to a counter for the recording of light-beam interruptions. Mice were placed individually in cages for 30 min, and the number of crossings of the light beams was recorded as the locomotor activity for 4 min.

#### 4.3.6. Rotarod Test

This experimental procedure was described in detail in our earlier studies [15,64]. Briefly, mice were trained on the rotarod apparatus (May Commat RR0711, Turkey; rod diameter: 2 cm) every day for 3 consecutive days. During each training session, mice were placed for 3 min on the rotating rod (24 rpm, constant speed) with an unlimited number of trials. The experiment was performed 24 h after the last training session. On the test day, mice were injected with HBK-10, and 30 min after the administration tested on the rotarod (24 rpm, constant speed). Motor impairment was regarded as the inability to remain on the rotating rod for 60 s and was expressed as the % of animals that fell off the rotating rod.

#### 4.3.7. Chimney Test

The chimney test was performed as previously described [60,68]. The mice were trained before the test, and only those animals able to get out of the chimney within 1 min, were used at the experimental stage. The selected mice were placed in a 25 cm long and 2.5 cm in diameter horizontally located tube, which was reversed so that the mice could leave it only by climbing up backward until they reached the other end. Motor impairment was indicated by the inability of the mice to perform the test within 60 s. The number of animals unable to climb backward within 60 s was recorded, and TD_50_ values were calculated.

#### 4.3.8. Corticosterone-Induced Mouse Model of Depression

**Figure 8:**
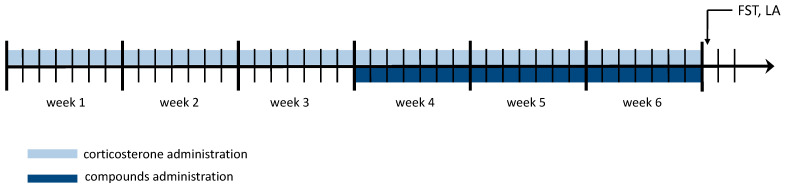
Schematic diagram of experimental design (corticosterone-induced model of depression) Corticosterone or vehicle were injected *sc* to mice for 3 weeks at random times during the light phase. During the next 3 weeks, 30 min before the corticosterone administration, mice were *ip* injected with HBK-10 (0.625 or 1.25 mg/kg), fluoxetine (15 mg/kg), or saline. 24 h after the last compounds’ administration, the behavioral tests were performed. FST—forced swim test; LA—locomotor activity.

Mice were injected with corticosterone (*sc*, 20 mg/kg) at random times during the light phase for 21 days. The dose and route of administration of corticosterone were based on our previous study [21]. Control mice received saline containing 0.1% dimethyl sulfoxide (DMSO) and 0.1% Tween (*sc*). For the next 21 days, 30 min before the corticosterone injection, mice were injected with saline, HBK-10 (0.625 or 1.25 mg/kg), or fluoxetine (15 mg/kg) (*ip*). Twenty-four hours after the last injection, animals were tested in the forced swim test and locomotor activity test (Figure 8).

### 4.4. Statistical Analysis

The number of animals in groups was based on our previous experiments [19,49,64]. The normality of data sets and their homogeneity were determined using Shapiro–Wilk and Brown–Forsythe test, respectively. Comparisons between experimental and control groups were performed by one- or two-way ANOVA, followed by Newman–Keuls post hoc. In cases when assumptions for normal distribution of data was not fulfilled (rotarod and chimney tests), we used Kruskal–Wallis with Dunn’s post hoc test.

## 5. Conclusions

This work presents the synthesis and preliminary pharmacological evaluation of a novel 2-methoxyphenylpiperazine derivative—HBK-10. We proved that HBK-10 is a 5-HT_1A_ and D_2_ antagonist and elicits the antidepressant-like effect in naïve and corticosterone-treated mice (model of depression). The observed behavioral effects are most likely due to the interaction with noradrenergic and serotonergic systems, particularly with the 5-HT_1A_ receptor. Overall, the present observations suggest that HBK-10 may be a model structure for future synthesis of compounds with potential use in depression treatment and therefore requires further investigation to unravel its precise mechanism of action.

## Figures and Tables

**Table 1:** Viability of HepG2 cells incubated with HBK-10 in concentration 1–25 μM for 24 h.

HBK-10 Concentration	Absorbance (OD570 nm)	Viability (% ± SEM)
Blank	0.516	100
1	0.531	102 ± 3.3
5	0.569	109 ± 0.9
25	0.463	99 ± 2.4

**Table 2:** Microsomal stability of HBK-10 and the reference drugs estimated in vitro in microsomal model (MLMs).

Treatment	Protein Concentration	% Remaining after 30 min	t_1/2_ min	Intrinsic Clearance (Cl_int_)
HBK-10	0.8	36	25	34.6
Buspirone	0.5	0.29 ^a^	-	-
Aripiprazole	1.0	-	217 ^b^	12.5 ^b^
Imipramine	0.5	-	11.0 ^c^	125.5 ^c^

^a^ literature data from [27]; ^b^ literature data from [25]; ^c^ literature data from [26].

**Table 3:** The affinity of HBK-10 for serotonergic 5-HT_1A_, 5-HT_2A_, 5-HT_6_, 5-HT_7_ receptors, dopaminergic D_2_ receptor, and serotonin transporter.

Treatment	Serotonergic p*K_i_* [nM]					Dopaminergic p*K_i_* [nM]
	5-HT_1A_^a,c^	5-HT_2A_^a,d^	SERT ^b,e^	5-HT_6_^a,f^	5-HT_7_^a,g^	D_2_^a,h^
HBK-10	8.29 ± 0.10	6.72 ± 0.31	5.43 ± 0.32	5.43 ± 0.22	5.20 ± 0.20	7.30 ± 0.19
Serotonin	8.62 ± 0.13	-	-	-	-	-
Mianserin	-	8.92 ± 0.06	-	-	-	-
Risperidone	-	9.70 ± 0.19	-	-	-	-
Escitalopram	-	-	8.68 ± 0.03	-	-	-
Clozapine	-	-	-	8.40 ± 0.13	7.74 ± 0.16	-
Haloperidol	-	-	-	-	-	8.96 ± 0.07

Data are represented as p*K_i_*, that is, −log*K_i_* and expressed as means ± SEM from three independent experiments performed in duplicates. Inhibition constants (*K_i_*) were calculated according to the equation of Cheng and Prusoff [28]. ^a^ Radioligand binding was performed using CHO-K1 cells. ^b^ Radioligand binding was performed using rat cortex tissue. ^c^ The affinity values were determined using [^3^H]8-OH-DPAT. ^d^ The affinity values were determined using [^3^H]-ketanserin. ^e^ The affinity values were determined using [^3^H]-citalopram. ^f^ The affinity values were determined using [^3^H]-LSD. ^g^ The affinity values were determined using [^3^H]-5-CT. ^h^ The affinity values were determined using [^3^H]-methylspiperon. SERT—serotonin transporter.

**Table 4:** Intrinsic activity of HBK-10 toward the serotonin 5-HT_1A_ receptor.

	Agonist Mode			Antagonist Mode		
Treatment	E_max_ %	pEC_50_ ± SEM	E_max_ %	pIC_50_ ± SEM	K_b_	R^2^
					nM	K_b_
Serotonin	100%	8.08 ± 0.36	-	-	-	-
NAN-190	-	-	2%	7.72 ± 0.114	6.4	0.919
HBK-10	2%	n.c.	0%	6.72 ± 0.093	64	0.831

Data are expressed as means ± SEM from three independent experiments performed in duplicates. E_max_, the maximum possible effect; pIC_50_, the logarithm of concentration of a compound where 50% of its maximal inhibitory effect was observed; K_b_, the equilibrium dissociation constant for a competitive antagonist determined using the Cheng–Prusoff equation; n.c., not calculable. The functional assay was performed using CHO-K1 cells.

**Table 5:** Intrinsic activity of HBK-10 at the dopamine D_2_ receptor.

	Agonist Mode			Antagonist Mode		
Treatment	E_max_ %	pEC_50_ ± SEM	E_max_ %	pIC_50_ ± SEM	K_b_ nM	R^2^ K_b_
Apomorphine	100%	7.89 ± 0.36	1%	n.c.	n.c.	n.c.
Chlorpromazine	5%	n.c.	1%	8.53	0.88	0.956
HBK-10	9%	n.c.	0%	8.03	2.76	0.985

Data are expressed as means ± SEM from three independent experiments performed in duplicates. E_max_, the maximum possible effect; pIC_50_, the logarithm of concentration of a compound where 50% of its maximal inhibitory effect was observed; K_b_, the equilibrium dissociation constant for a competitive antagonist determined using the Cheng–Prusoff equation; n.c., not calculable. The functional assay was performed using CHO-K1 cells.

**Table 6:** The effect of HBK-10 and fluoxetine on locomotor activity in corticosterone-treated mice after a chronic administration.

Treatment	Corticosterone Injections	Dose (mg/kg)	Number of Crossings ± SEM
vehicle	-	-	515.9 ± 41.18
vehicle	+	-	402.9 ± 43.30
HBK-10	+	0.625	431.1 ± 47.92
HBK-10	+	1.25	453.9 ± 41.27
Fluoxetine	+	15	462.5 ± 41.81

After 3 weeks of either corticosterone (20 mg/kg) or vehicle (saline containing 0.1% dimethyl sulfoxide (DMSO) and 0.1% Tween) injections (subcutaneously (*sc*)), mice were administered intraperitoneally (*ip*) with HBK-10 (0.625 or 1.25 mg/kg), fluoxetine (15 mg/kg), or vehicle (1% Tween) for next 3 weeks, 30 min before the corticosterone or vehicle injections. 24 h after the last compounds’ administration, the locomotor activity was recorded individually for each animal in activity cages. After habituation time (30 min), the number of crossings of photobeams was measured during the next 4 min. The results are expressed as mean ± SEM. Statistical analysis: Shapiro–Wilk test for normality, Brown–Forsythe test for homogeneity of variance, and one-way ANOVA (Newman–Keuls post hoc); *n* = 8 mice per group.

**Table 7:** The effect of HBK-10 on locomotor activity in mice.

Treatment	Dose (mg/kg)	Number of Crossings ± SEM	ED_50_ (mg/kg)
vehicle	-	355.1 ± 31.38	32.3 (19.2–54.3)
HBK-10	10	355.1 ± 49.92	
	20	329.8 ± 34.52	
	40	155.4 ± 29.59 ***	
	60	22.17 ± 2.03 ****	

The locomotor activity was recorded individually for each animal in activity cages. After habituation time (30 min) the number of crossings of photobeams was measured during next 4 min. The tested compound or vehicle (1% Tween) were administered *ip* 30 min before the test. The results are expressed as means ± SEM. Statistical analysis: Shapiro–Wilk test for normality, Brown–Forsythe test for homogeneity of variance, and one-way ANOVA (Newman–Keuls *post hoc*); *** *p* < 0.001, **** *p* < 0.0001; *n* = 8–10 mice per group.

**Table 8:** The effect of HBK-10 on motor coordination in rotarod and chimney test in mice.

Rotarod Test				Chimney Test		
Treatment	Dose (mg/kg)	% of Animals That Fell from Rotating Rod	TD_50_ (mg/kg)	Dose (mg/kg)	% of Animals That Did Not Climb out the Chimney	TD_50_ (mg/kg)
HBK-10			44.3 (37.8–51.8)	10	20	21.7 (13.0–36.4)
	40	29		20	40	
	45	63		30	64	
	60	86		40	87	
				60	90	

Rotarod: After three days of training, the studied compound was administered *ip* 30 min before the test. The results are presented as the percentage of animals that could not stay on a rotating rod for at least 1 min. Chimney test: After a training session, the studied compound was injected *ip* 30 min before the test. The results are presented as the percentage of animals that did not climb out after 1 min. *n* = 8–10 animals per group. TD_50_ values were calculated using the log-probit method described by Litchfield and Wilcoxon [37].

**Table 9:** Radioligand binding assays conditions.

Receptor	Radioligand/Final Concentration	Blank (Nonspecific)	Buffer	Incubation Conditions
5-HT_1A_	[^3^H]8-OH-DPAT 1 nM	10 µM serotonin	50 mM Tris-HCl pH 7.4 10 mM MgSO_4_, 0.5 mM EDTA, 0.1% ascorbic acid	60 min, 27 °C
5-HT_2A_	[^3^H]-ketanserin 0.5 nM	10 µM mianserin	50 mM Tris-HCl pH 7.4 4 mM CaCl_2_, 0.1% ascorbic acid	60 min, 27 °C
5-HT_6_	[^3^H]-LSD 2 nM	10 µM methiothepin	50 mM Tris–HCl pH 7.4 0.5 mM EDTA, 4 mM MgCl_2_	60 min, 37 °C
5-HT_7_	[^3^H]-5-CT 0.6 nM	10 µM serotonin	50 mM Tris–HCl pH 7.4 4 mM MgCl_2_, 10 µM pargyline, 0.1% ascorbic acid	60 min, 37 °C
SERT	[^3^H]-citalopram 1 nM	1 µM imipramine	50 mM Tris-HCl pH 7.7 150 mM NaCl, 5 mM KCl	60 min, 23 °C
D_2_	[^3^H]-methylspiperon 0.4 nM	10 µM (+)-butaclamol	50 mM HEPES, pH 7.4, 50 mM NaCl, 5 mM MgCl2, 0.5 mM EDTA	60 min, 37 °C

## Data Availability

Data is contained within the article.
